# Behavioral Phenotyping and Pathological Indicators of Parkinson's Disease in *C. elegans* Models

**DOI:** 10.3389/fgene.2017.00077

**Published:** 2017-06-13

**Authors:** Malabika Maulik, Swarup Mitra, Abel Bult-Ito, Barbara E. Taylor, Elena M. Vayndorf

**Affiliations:** ^1^Department of Chemistry and Biochemistry, University of Alaska FairbanksFairbanks, AK, United States; ^2^Department of Biology and Wildlife, University of Alaska FairbanksFairbanks, AK, United States; ^3^Department of Biological Sciences, California State University, Long BeachLong Beach, CA, United States; ^4^Institute of Arctic Biology, University of Alaska FairbanksFairbanks, AK, United States

**Keywords:** Parkinson's disease (PD), *Caenorhabditis elegans* (*C. elegans*), behavioral phenotyping, dopamine, pathological markers

## Abstract

Parkinson's disease (PD) is a neurodegenerative disorder with symptoms that progressively worsen with age. Pathologically, PD is characterized by the aggregation of α-synuclein in cells of the substantia nigra in the brain and loss of dopaminergic neurons. This pathology is associated with impaired movement and reduced cognitive function. The etiology of PD can be attributed to a combination of environmental and genetic factors. A popular animal model, the nematode roundworm *Caenorhabditis elegans*, has been frequently used to study the role of genetic and environmental factors in the molecular pathology and behavioral phenotypes associated with PD. The current review summarizes cellular markers and behavioral phenotypes in transgenic and toxin-induced PD models of *C. elegans*.

## Introduction

Parkinson's disease (PD) is the second most prevalent age-related neurodegenerative disorder after Alzheimer's disease. It affects seven to 10 million individuals worldwide (Beitz, 2014), with the mean age of onset at 60 years, where 1% of all individuals over the age of 60 and 4% of those over 80 years present with PD symptoms. PD is characterized by the progressive loss of dopaminergic neurons in the substantia nigra pars compacta (nigrostriatal pathway) area of the brain (Michel et al., 2013; Kalia and Lang, 2015). At the cellular level, the hallmarks of PD include intra-cytoplasmic inclusions that contain a disease-specific protein: α-synuclein, a primary component of Lewy bodies and dystrophic Lewy neurites in neurons (Bethlem and Den Hartog Jager, 1960; Spillantini et al., 1997; Dickson, 2012). The loss of dopaminergic neurons results in motor impairments, including tremors, hypokinesia, bradykinesia, rigidity, and postural instability (Samii et al., 2004; Jankovic, 2008; Yao S. C. et al., 2013). Other recognizable motor deficits include festination, speech and swallowing disorders, and handwriting in small letters (Jankovic, 2008; Russell et al., 2010). Since PD affects neurons in the central and peripheral nervous systems, patients typically also exhibit multiple non-motor symptoms including anxiety, depression, memory loss, and olfactory deficits (Doty, 2012; Grover et al., 2015). While the cause of PD is currently unknown, genetic (familial) and environmental (sporadic) triggers are two major factors that play a role in the development of the disease, with the environment accounting for over two-thirds of all cases (Fleming et al., 1994; Warner and Schapira, 2003; Gatto et al., 2010; Goldman et al., 2012; Trinh and Farrer, 2013). The predisposition to both sporadic and familial types of PD is linked to multiple genes whose function is an area of active investigation. These include α-synuclein, LRRK2, PARK2, *DJ-1*, GBA, UCHL1 and others. For example, a mutation in the glucocerebrosidase (GBA) gene, which codes for an enzyme essential for metabolism of lysosomal substrates is linked to the pathogenesis of sporadic PD (Gegg et al., 2012). Similarly, a mutation in the ubiquitin carboxyl-terminal hydrolase L1 (UCHL1), an enzyme which is involved in the removal and recycling of ubiquitin molecules from degraded proteins, and ligation of ubiquitin to proteins to mark them for degradation, has been linked to the early-onset of familial PD (Dawson and Dawson, 2003). The identification of these and other genes, and the discovery that certain toxins such as MPTP, 6-OHDA, and paraquat lead to PD symptoms, has informed the development of genetic and toxin-induced PD models (Polymeropoulos et al., 1997; Bonifati et al., 2003; Paisán-Ruiz et al., 2004; Valente et al., 2004; Zimprich et al., 2004) and resulted in a better understanding of disease etiology, pathology, and molecular mechanisms (Harrington et al., 2010; Whitworth, 2011; Blesa et al., 2012a).

In mammalian models, genetically modified rodents have proven critical to the understanding of PD pathology and the exploration of new therapeutic strategies (Ribeiro et al., 2013). Rodent models display many of the clinical features of PD such as the loss of dopaminergic neurons (Meredith and Rademacher, 2011; Thiele et al., 2012; Torres and Dunnett, 2012), neurochemical changes in dopamine transmission and signaling, motor dysfunction, and non-motor symptoms including cognitive decline, autonomic dysfunction, depression, and hyposmia (Taylor et al., 2010; Schirinzi et al., 2016). However, these models do not mimic some important pathological hallmarks of the disease (Fleming and Chesselet, 2006; Visanji et al., 2016) such as the gradual neurodegenerative process, gross morphological abnormalities and overt motor alterations (Yue and Lachenmayer, 2011; Ribeiro et al., 2013; Schirinzi et al., 2016). Moreover, gene editing techniques in rodents involve complex experimental design, significant time investment and considerable expense. Environmental toxin-induced rodent models have also provided valuable information about PD pathology. For example, 1-methyl-4-phenyl-1,2,3,6-tetrahydropyridine (MPTP) induces a severe and permanent parkinsonism syndrome that features the major symptoms of human PD including rigidity, tremor, postural instability, and slowness of movement (Liou et al., 1997; Bove et al., 2005). In addition, environmental exposure to paraquat is a risk factor for PD; paraquat administration increases α-synuclein levels and α-synuclein-positive inclusion bodies in substantia nigra neurons (Manning-Bog et al., 2002). A major drawback of toxin-induced models is that acutely induced neurodegeneration investigates a phase of PD when nearly 70–80% of dopaminergic neurons are already lost, thus lacking the age-dependent progressive lesions and Lewy bodies that are typical of human patients (Blandini and Armentero, 2012; Schirinzi et al., 2016).

Non-mammalian, including invertebrate models such as *Drosophila melanogaster* and *Caenorhabditis elegans* are also useful in understanding the molecular mechanisms of PD (Jagmag et al., 2015). These models facilitate investigations of PD-associated molecular signaling pathways and first-round screening that can be followed-up in mammalian models (Jagmag et al., 2015). For example, *D. melanogaster* transgenic models have helped clarify the role of PD candidate genes in mitochondrial physiology (Venderova et al., 2009; Dawson et al., 2010; Guo, 2012). Similarly, the nematode *C. elegans* is a useful model organism for studying healthy and abnormal neuronal aging, including cellular symptoms of PD. *Caenorhabditis elegans* share many conserved cellular pathways and mechanisms with mammals, including humans (Consortium, 1998; Lai et al., 2000; Shaye and Greenwald, 2011). These cellular pathways can be genetically manipulated using RNA interference (RNAi) by gene-specific bacterial feeding (Fire et al., 1998), which enables rapid screening of target genes (Jorgensen and Mango, 2002; Wang and Sherwood, 2011). RNAi screening is an important tool for predicting pathogenic mechanisms before moving to complex organisms for further investigation (Jorgensen and Mango, 2002; Leung et al., 2008; O'Reilly et al., 2014). Despite major anatomical differences from humans, the *C. elegans* nervous system consists of a circumpharyngeal nerve ring, and contains key cellular and molecular features of mammalian neurons, including conserved neurotransmitter systems (dopamine, GABA, acetylcholine, serotonin, etc.), receptors, axon guidance molecules, ion channels, and synaptic features. Although α-synuclein is not endogenous to *C. elegans*, expression of this human PD-associated protein in *C. elegans* dopaminergic neurons results in neurodegeneration in an age-dependent manner (Lakso et al., 2003; Kuwahara et al., 2006; Hamamichi et al., 2008; Karpinar et al., 2009). Moreover, most familial PD genes such as *PINK1, PARK, DJ-1*, and *LRRK2* have at least one *C. elegans* homolog (Sakaguchi-Nakashima et al., 2007; Sämann et al., 2009; Chege and Mccoll, 2014; Lee and Cannon, 2015). Hermaphroditic *C. elegans* have 302 neurons, of which eight (ADEL, ADER, CEPDL, CEPDR, CEPVL, CEPVR, PDEL, and PDER) are dopaminergic such as those implicated in PD in humans (Sulston et al., 1975). Four dopamine receptors (DOP-1, DOP-2, DOP-3, and DOP-4) have been identified in *C. elegans*, including homologs of each of the two classes of mammalian dopamine receptors (D1- and D2-like) (Chase and Koelle, 2007). *Caenorhabditis elegans* neuronal morphology can be linked to functional abnormalities for easy visualization and quantification making it possible to establish a correlation between behaviors and aberrations in the target neurons, which are induced by mutations or exposure to toxins (Nass et al., 2002; Toth et al., 2012; Scerbak et al., 2014; Vayndorf et al., 2016). In addition, *C. elegans* have low maintenance costs, and their shorter lifespan (2–3 weeks) reduces the time needed for each experiment. These advantages make *C. elegans* a valuable model system for genetic and chemical screening, and pre-clinical research. In contrast, the limitations of a *C. elegans* PD model include a lack of defined organs, including the complex brain structure seen in humans and, therefore, the inability to recapitulate the same set of complex interactions involving various brain cells and tissues seen in human PD patients (Tissenbaum, 2015). In addition, the mostly impermeable cuticle and inability of intestinal cells to take up some types of chemicals may require high exposure doses to affect the animal's physiology (Leung et al., 2008; Tissenbaum, 2015). Despite these limitations, *C. elegans* have proven useful in aging research (Tissenbaum, 2015) and numerous studies have used *C. elegans* to investigate the cellular mechanisms associated with PD (see Table 1). The aim of this review is to highlight the genetic and chemical tools and reagents, as well as genetic, biochemical, physiological, and behavioral endpoints associated with investigating the cellular and behavioral symptoms of PD in *C. elegans*.

**Table 1:** Strains of *C. elegans* commonly used to study PD pathology.

**Strain Name**	**Genotype**	**Available through the CGC?**	**Pathological markers**	**Behavioral phenotype**	**References**
N2	Wild isolate Bristol	Yes	Estimation of DA content post treatment with MPTP/6-OHDA/insecticides and compounds.	Not reported.	Ali and Rajini, 2012; Fu et al., 2014a; Chen P. et al., 2015; Satapathy et al., 2016
			Not reported.	Locomotion.	Ali and Rajini, 2012; Li J. et al., 2016; Xu et al., 2017
				Fecundity.	Fitsanakis, 2012; Nidheesh et al., 2016; Satapathy et al., 2016
NL5901	unc-54p::α-synuclein::YFP + unc-119(+)	Yes	1. α-synuclein overexpression.	Not reported.	van Ham et al., 2008; Jadiya et al., 2011, 2012; Bodhicharla et al., 2012; Jadiya and Nazir, 2012; Jensen et al., 2012; Shukla et al., 2012; Shi et al., 2013; Fatima et al., 2014; Munoz-Lobato et al., 2014; Sashidhara et al., 2014; Cooper et al., 2015; Edwards et al., 2015; Heiner et al., 2015; Liu et al., 2015; Asthana et al., 2016; Li J. et al., 2016; Xu et al., 2017
			2. Estimation of Lipid content using Nile red.		Jadiya et al., 2011; Jadiya and Nazir, 2012
			Not done.	Chemotaxis (Nonanol repulsion assay).	Fatima et al., 2014; Sashidhara et al., 2014
OW13	unc-54p::α-synuclein::YFP + unc-119(+)	Yes	1. α-synuclein overexpression.	Not reported.	van Ham et al., 2008; Fu et al., 2014a,b; Chen Y. M. et al., 2015
			2. Estimation of Lipid content using Nile red.	Not reported.	Fu et al., 2014a,b; Chen Y. M. et al., 2015
DDP1	uonEx1 [unc-54::alpha-synuclein::CFP + unc-54::alpha-synuclein::YFP(Venus)]	Yes	Monitoring the influence of genetic and/or environmental factors on the extent of α-synuclein aggregation using FRET signals.	Reduced lifespan, reduced pharyngeal pumping compared to N2.	Bodhicharla et al., 2012
BZ555	dat-1p::GFP	Yes	Dopaminergic degeneration using agents such as 6-OHDA, MPTP, insecticide like monocrotophos.	Not reported.	Pu and Le, 2008; Jadiya et al., 2011; Ali and Rajini, 2012; Fitsanakis, 2012; Fatima et al., 2014; Fu et al., 2014a; Chen Y. M. et al., 2015; Li J. et al., 2016; Nidheesh et al., 2016; Satapathy et al., 2016; Xu et al., 2017
			Not reported.	Basal response to food.	Li J. et al., 2016
JVR105	cwrIs730 [dat-1p::GFP, lin-15(+)]	No	Neuronal morphology.	1. Basal slowing. 2. Ethanol avoidance. 3. Area restricted searching.	Cooper et al., 2015
BY200	dat-1p::GFP, pRF4(rol-6(su1006)	No	Dopaminergic degeneration using agents such as 6-OHDA, MPTP.	Locomotion.	Nass et al., 2002; Benedetto et al., 2010; Settivari et al., 2013; Masoudi et al., 2014
BY250	dat-1p::GFP	No	Dopaminergic degeneration using agents such as 6-OHDA, Manganese, Uranium, bacterial metabolite, methyl mercury, aluminum.	Not reported.	Nass et al., 2005; Jiang et al., 2007; Settivari et al., 2009; VanDuyn et al., 2010, 2013; Zhou et al., 2013; Gonzalez-Hunt et al., 2014; Ray et al., 2014
JVR103	dat-1p::GFP	No	Dopaminergic degeneration.	1. Basal response to food.	Cooper et al., 2015
				2. Locomotion.	
				3. Area restricted searching.	
				4. Ethanol avoidance.	
UA57	dat-1p::GFP + dat-1p::CAT-2	Yes	1. Dopaminergic degeneration using MPTP.	Not reported.	Braungart et al., 2004; Yao et al., 2010a; Masoudi et al., 2014; Liu et al., 2015
			2. Age dependent dopaminergic degeneration.		
UA44	dat-1p::α-synuclein+dat-1p::GFP	No	α-synuclein-induced DA neuronal death.	Not reported.	Cao et al., 2005; Buttner et al., 2014; Munoz-Lobato et al., 2014
BY273	dat-1p::GFP; dat-1p::WTα-synuclein	No	Dopaminergic degeneration induced by α-synuclein using manganese and aluminum.	Not reported.	Settivari et al., 2009; VanDuyn et al., 2013
JVR107 (Strain name not published for Kuwahara et al., 2006, 2008)	dat-1p:: α-synuclein[A53T]	No	Dopaminergic degeneration.	1.Basal response to food.	Cooper et al., 2015
				2. Locomotion.	
				3. Area restricted searching.	
				4. Ethanol avoidance.	
			1. Dopaminergic degeneration.	Basal response to food.	Kuwahara et al., 2006, 2008
			2. Estimation of DA content.		
JVR203	dat-1p::α-synuclein[A53T]; vtIs7[dat-1p::GFP(pRB490)]	No	Dopaminergic degeneration.	1. Basal response to food.	Cooper et al., 2015
				2. Locomotion.	
				3. Area restricted searching.	
				4. Ethanol avoidance.	
JVR104	cwrIs856 [dat-1p::GFP, datp-1::LRRK2(WT), lin-15(+)], cwrIs722 [dat-1p::GFP, Pdat-1::LRRK2 (WT), lin-15(+)]	No	Dopaminergic degeneration.	1. Basal response to food.	Cooper et al., 2015
JVR168				2. Locomotion.	
SGC722				3. Area	
				restricted searching.	
				4. Ethanol avoidance.	
			1. Age-dependent degeneration of DA neurons due to overexpression of LRRK2 (WT).	1. Basal response to food.	Yao et al., 2010a
			2. Estimation of DA content.	2. Locomotion.	
Not published	dat-1p:: α-synuclein	No	Dopaminergic degeneration.	Not reported.	Cao et al., 2005; Kautu et al., 2013
Not published	dat-1p:: α-synuclein[A53T]	No	Dopaminergic degeneration.	Locomotion.	Lakso et al., 2003
SGC851	lin-15(n765ts) X; cwrIs851 [dat-1p::GFP, dat-1p::LRRK2(R1441C), lin-15(+)]	No	1. Age-dependent degeneration of DA neurons due to overexpression of LRRK2 (R1441C).	1. Basal response to food.	Yao et al., 2010a; Yao C. et al., 2013
			2. Estimation of DA content.	2. Locomotion.	
SGC856	lin-15(n765ts) X; cwrIs856 [dat-1p::GFP, dat-1p::LRRK2(G2019S), lin-15(+)]	No	1. Age-dependent degeneration of DA neurons due to overexpression of LRRK2 (G2019S).	1. Basal response to food.	Yao et al., 2010a; Yao C. et al., 2013
			2. Estimation of DA content.	2. Locomotion.	
MAB147	(mjaEx109) [djr-1.1p::GFP; rol6(su1006)]	No	Not reported.	Dauer dependant behavior.	Chen P. et al., 2015
MAB82	(mjaEx050 [djr-1.2p::GFP; Rol 6(su1006)]; otls181)	No	Not reported.	Dauer dependant behavior.	Chen P. et al., 2015
BR3646, BR3645	(pha-1(e2123);byEx686[pink-1]),(pha-1(e2123);byEx687[pink-1])	No	Mitchondrial homeostasis and oxidative stress response.	Fecundity.	Sämann et al., 2009
Not published	dat-1p::GFP + dat-1p::α–synco-expressed with dat-1p::FLAG-W08D2.5	No	Age-dependent degeneration of DA neurons due to over-expression of α-synuclein.	Not reported.	Gitler et al., 2009
VC1024	pdr-1 (gk448) III	Yes	Not done.	Basal response to food.	Martinez-Finley et al., 2013

## *Caenorhabditis elegans* models of Parkinson's disease

In this section, we discuss the link between genetic and environmental factors and PD. All existing *C. elegans* models are the result of genetic manipulation or exposure to toxic chemicals.

### Genetic *C. elegans* models linked to familial PD

Over the last decade, transgenic models of *C. elegans* have been successfully used to study PD-like pathologies and behaviors (Caldwell and Caldwell, 2008; Harrington et al., 2010). In humans, monogenic forms of PD, caused by a single gene mutation in a dominant or recessive fashion, are well-established, though relatively rare types of the disease. They account for approximately 30% of the familial cases (Klein and Westenberger, 2012).

#### Alpha-synuclein

Alpha-synuclein is a small, highly soluble, predominantly presynaptic cytoplasmic protein composed of 140 amino acids with three domains. It is highly conserved in vertebrates and has been implicated in PD and other synucleinopathies (Snead and Eliezer, 2014). In humans, α-synuclein is largely present in the brain, with smaller amounts also present in the heart, muscles, and other tissues (Xu and Pu, 2016). While the normal physiological structure and function of α-synuclein is unclear, studies suggest that it is important for compartmentalization, storage, and recycling of neurotransmitters (Lee et al., 2002). In addition, α-synuclein can regulate a variety of enzymes, is thought to increase the number of dopamine transporters, and has molecular chaperone activity, which is linked to neurotransmitter release (Nemani et al., 2010). The α-synuclein gene, *SNCA*, is causatively related to PD and its mutation was the first gene to be linked to the disease (Polymeropoulos et al., 1997). Mutations in *SNCA*, including rare point mutations in the N-terminal domain of α-synuclein as well as duplications and triplications of wild-type α-synuclein cause familial forms of PD in humans (Ross et al., 2008; Klein and Westenberger, 2012; Singleton et al., 2013).

*Caenorhabditis elegans* do not have an α-synuclein homolog. Thus, to study the pathogenicity of α-synuclein overexpression and aggregation in PD, several transgenic *C. elegans* strains with human α-synuclein have been created. These strains are particularly useful for studying the toxicity of protein aggregates, and cellular and behavioral abnormalities (Hamamichi et al., 2008; van Ham et al., 2008). Strains OW13 ([unc-54p::α-synuclein::YFP + unc-119(+)]), NL5901 ([unc-54p::α-synuclein::YFP+unc-119(+)]), and DDP1 (uonEx1[unc-54p::α-synuclein::CFP + unc-54::α-synuclein::YFP(Venus)] express α-synuclein in body wall muscle cells (van Ham et al., 2008; Bodhicharla et al., 2012). In these strains, the human α-synuclein gene is fused to yellow fluorescent protein (YFP), which drives the expression of α-synuclein in the body wall muscle cells under the control of the *unc-54* promoter (Hamamichi et al., 2008; van Ham et al., 2008; Bodhicharla et al., 2012). These strains have been used to study α-synuclein aggregation, changes in movement, animal behavior and genes that modulate these and other PD-related hallmarks. For example, the brains of PD patients contain electron-dense filamentous and granular protein inclusions filled with aggregated protein. Similarly, *C. elegans* body wall muscle cells accumulate clearly visible aggregates with age, providing a defined target for screening of candidate genes via RNAi. Van Ham and colleagues have identified 80 suppressors of inclusion formation, with 49 of these genes having an established human ortholog. These authors also found an increase in the number of “immobile” inclusions relative to “mobile” inclusions during aging (van Ham et al., 2008). The accumulation of α-synuclein aggregates in these strains is associated with locomotory and movement impairments (Bodhicharla et al., 2012) providing additional screening targets. All three strains containing α-synuclein in body wall muscle cells are available from the *Caenorhabditis* Genetics Center at the University of Minnesota for a nominal shipping charge (see Table 1).

In addition to strains that overexpress α-synuclein in body wall muscle cells, strains that overexpress wild-type or mutant (A53T) human α-synuclein in dopaminergic neurons have been generated by multiple research groups (Lakso et al., 2003; Kuwahara et al., 2006, 2008; Cooper et al., 2015). In these models, the dopamine transporter promoter *dat-1* is fused to GFP, following co-expression of wild-type or mutant (A53T) α-synuclein and GFP. The A53T mutation causes a change from alanine to threonine at position 53, is highly penetrant, and is associated with the autosomal dominant form of PD (Polymeropoulos et al., 1997; Lakso et al., 2003). In *C. elegans* expressing both wild-type and mutant α-synuclein, neuronal abnormalities, including accumulation of aggregates and cell loss were observed in some or all dopaminergic neurons, typically in an age-dependent manner (Lakso et al., 2003; Kuwahara et al., 2006, 2008; Cooper et al., 2015). Moreover, neurodegeneration of dopamine neurons was enhanced in transgenic lines in which mRNA levels of α-synuclein were expressed at higher levels (Dexter et al., 2012).

In humans, fibrils of α-synuclein aggregate to form Lewy bodies, intracellular inclusions of protein complexes made of α-synuclein aggregates and other components such as neurofilaments, lipids and membrane materials (Spillantini et al., 1997; van Ham et al., 2008). Lewy bodies are a major hallmark of PD. When human α-synuclein is expressed in *C. elegans* dopaminergic neurons, expression as inclusion bodies is rare and aggregation of α-synuclein is not observed in Western blots (Lakso et al., 2003). However, α-synuclein misfolding can be followed in body wall muscle cells as translational fusion YFP inclusions. In strains NL5901, OW13, and DDP1, which express these inclusions, α-synuclein aggregates and leads to toxicity with age. Importantly, large-scale reverse genetic RNAi screens have revealed enhancers and suppressors of α-synuclein misfolding, including genes that protect against α-synuclein neurodegeneration when co-expressed with α-synuclein in dopaminergic neurons (Hamamichi et al., 2008; van Ham et al., 2008).

#### LRK-1 and PINK-1

In PD patients, mutations in the multi-domain protein leucine-rich repeat kinase 2 (LRRK2) are the most common genetic risk factors for both familial and sporadic PD, accounting for 4% of familial and 1% of sporadic PD across all populations (Healy et al., 2008). Mutations are prevalent within the GTPase (R1441C/G) and kinase (G2019S) domains of LRRK2. The normal function of LRRK2 is an area of active investigation, with research suggesting remarkably diverse pathways including regulation of transcription (Kanao et al., 2010), translation (Imai et al., 2008), apoptosis (Ho et al., 2009), and mitochondrial function (Smith et al., 2005). LRRK2 is consistently located at intracellular membranous structures including mitochondria (West et al., 2005; Biskup et al., 2006; Gloeckner et al., 2006; Hatano et al., 2007), the endo-lysosomal system (Alegre-Abarrategui et al., 2009), the endoplasmic reticulum (ER) (Gloeckner et al., 2006; Vitte et al., 2010), and Golgi *C. elegans* (Biskup et al., 2006; Gloeckner et al., 2006; Hatano et al., 2007).

In *C. elegans*, the *lrk-1* gene is homologous to mammalian *LRRK1* and *LRRK2*, human and mouse leucine-rich repeat kinases, respectively. LRRK1 is necessary for polarized localization of synaptic vesicle proteins to presynaptic regions (Shin et al., 2008; Esposito et al., 2012). LRK-1 is expressed in many tissues, including head and tail neurons, hypodermis, intestine and muscles, and localizes to the Golgi apparatus (Sämann et al., 2009).

Two types of *C. elegans* genetic models have been used to study the leucine-rich repeat kinase and its contribution to PD-like symptoms. In the first, two *lrk-1* mutant strains that each contain severe loss-of-function alleles (*tm1898)* and (*km41*) that express truncated LRK-1 proteins consisting of the N-terminal ankyrin repeat, have been used to study *pink-1*, a PTEN-induced kinase and homolog of the PD-related human *PINK1*. Both alleles of *lrk-1* suppressed the paraquat sensitivity of *pink-1*(*tm1779*) mutants to restore survival to wild-type levels (paraquat toxicity is detailed in the Insecticides and Herbicides subsection of the Toxin-Induced Models section below). *Lrk-1*(*tm1898*) also suppressed the mitochondrial cristae defects of *pink-1*(*tm1779*) animals to wild-type levels suggesting that genetic deletion of *lrk-1* could compensate for both the oxidative stress sensitivity and the mitochondrial integrity observed in a *pink-1* loss-of-function allele. Interestingly, both *C. elegans lrk-1* allele mutants are not sensitive to paraquat and have an intact mitochondrial cristae, but exhibit an enhanced sensitivity to ER stress that can be rescued by *pink-1(tml779)*. Moreover, both *lrk-1* mutations suppressed *pink-1(tml779)*-mediated axon guidance defects suggesting that LRK-1 and PINK-1 act antagonistically in stress response and neurite outgrowth (Sämann et al., 2009). These results link *pink-1*/PINK1 and *lrk-1*/LRRK2 function to the pathological processes involved in PD, and highlight stress sensitivity and cytoskeletal defects as factors that may contribute to the onset of PD.

In the second approach, human wild-type and mutant G2019S and R1441C LRRK2 have been overexpressed in dopaminergic neurons of *C. elegans* under the expression of the dopamine transporter *dat-1* promoter co-injected with dat-1p::GFP to generate [dat-1p::GFP, dat-1p::LRRK2(WT), lin-15(+)] and [dat-1p::GFP, dat-1p::LRRK2(G2019S), lin-15(+)] (Yao et al., 2010b; Yao S. C. et al., 2013; Cooper et al., 2015). Overexpression of these LRRK2 proteins caused age-dependent degeneration of dopaminergic neurons, behavioral deficits, locomotory dysfunction, and reduced dopamine levels in transgenic models of *C. elegans*. In comparison to the overexpression of wild-type LRRK2, R1441C and G2019S mutants showed more severe phenotypes. Treatment with exogenous dopamine rescued the LRRK2-induced behavioral and locomotory deficits (Yao et al., 2010b; Yao S. C. et al., 2013).

#### PDR-1

Some autosomal recessive forms of PD are associated with mutations in PARKIN (*PARK2)*, an E3 ubiquitin ligase that is important for neuronal protein homeostasis (Lücking et al., 2000; Bonifati et al., 2003; Valente et al., 2004; Trempe and Fon, 2013). In *C. elegans*, the *PARK2* homolog *pdr-1* is an essential component in the degradation machinery during the response to proteotoxic stressors (Springer et al., 2005). Specifically, *pdr-1* was shown to play a role in the UPR pathway, and co-expression of mutant α-synuclein A53T and truncated *pdr-1* exacerbated mutant α-synuclein-induced toxicity in a UPR-independent way (Springer et al., 2005). Previously, Morimoto and colleagues showed that heat shock proteins and molecular chaperones play an important role in maintaining protein homeostasis (Morimoto et al., 1997; Morimoto, 2008). Failure of these proteins to prevent misfolding and clearance of toxic aggregated proteins disrupts protein homeostasis and contributes to aging in *C. elegans* (Satyal et al., 2000; David et al., 2010). Conversely, overexpression of chaperones can improve proteostasis and reduce aggregation in protein misfolding diseases (Calamini et al., 2011). Recently, a new proteostasis mechanism of protein clearance for toxic, misfolded, and aggregated proteins in *C. elegans* neurons was proposed by Melentijevic et al. (2017). In this model, extracellular vesicles called exophers pinch off from the soma of some types of neurons to jettison toxic protein aggregates and damaged organelles including mitochondria and lysosomes for downstream degradation. The authors note that fluorescently-labeled touch receptor neurons of animals that have a *pdr-1(gk448)* mutant genetic background or those treated with *pink-1* RNAi produce significantly more exophers than animals of a wild-type background (Melentijevic et al., 2017). These observations suggest that impaired mitochondrial genes linked to PD can increase exopher production and provide a potential new area of investigation for cellular hallmarks of PD (Melentijevic et al., 2017).

#### DJR-1.1 and DJR-1.2

In humans, the *DJ-1* gene is causally linked to familial PD (Bonifati et al., 2003). First identified as an oncogene (Nagakubo et al., 1997), its functions include transcriptional regulation, antioxidant activity (in particular after toxic insults), chaperone activity, protease cleavage, and mitochondrial regulation. DJ-1 activity is regulated by its oxidative status and excess oxidation renders the protein inactive, a hallmark observed in patients with sporadic and familial PD as well as some patients with Alzheimer's disease (Choi et al., 2006). DJ-1 can also act as a stress sensor and its expression is increased with stresses such as oxidative stress (Ariga et al., 2013). *C. elegans* have two *DJ-1* orthologs: *djr-1.1*, and *djr-1.2*; both encode a type of glyoxylase. This enzyme facilitates the removal of α-oxoaldehydes, byproducts of glucose oxidation, lipid peroxidation and DNA oxidation, which can react non-enzymatically with amino groups of proteins to form advanced glycation end-products (AGEs), which are linked to PD (Lee et al., 2012). DJR-1.2 localizes to the cytosol and is expressed throughout life in a variety of cell and tissue types such as head neurons (including dopaminergic neurons), pharyngeal muscle, the ventral nerve cord, spermatheca, excretory canal cells, and coelomocytes. Manganese (Mn) (discussed in more detail in the Manganese subsection of the Toxin-Induced Models section below) is an essential nutrient needed for protein and energy metabolism, metabolic regulation, protection from reactive oxygen species (ROS), and enzymes function. Environmental exposure to large doses of Mn can lead to manganism, which shares multiple features with PD and is an established risk factor for PD occurrence (Aschner et al., 2009). Previously, Benedetto and colleagues have shown that intracellular dopamine can lead to Mn-induced dopaminergic neurodegeneration in *C. elegans*, and that this process depends on a functional dopamine-reuptake transporter (DAT-1) and is associated with elevated oxidative stress and reduced lifespan (Benedetto et al., 2010). Neuronal expression of DJR-1.2 in the head and ventral nerve chord neurons is elevated after exposure to acute Mn (Chen P. et al., 2015) and *djr-1.2* is protective against Mn-induced dopaminergic toxicity in an age-dependent manner (Chen P. et al., 2015). Specifically, deletion of *djr-1.2* decreases survival and dopamine-dependent dauer movement behavior after Mn exposure, and lifespan could be rescued by overexpression of *djr-1.2* or *daf-16* (Chen P. et al., 2015) mitigating Mn-dependent lifespan reduction and dopamine signaling alterations, involving DAF-2/DAF-16 signaling. The *C. elegans djr-1.1*, also orthologous to *DJ-1*, localizes to the intestine and plays a primary role in protecting animals from glyoxal. Treatment of *djr-1.1*, and to a lesser extent *djr-1.2* deletion animals with glyoxal significantly improved their survival suggesting that this gene can protect animals from glyoxal-induced death (Lee et al., 2012).

#### DAT-1 and CAT-2

The human dopamine transporter (DAT) pumps dopamine out of the synapse back into the cytosol where other transporters deliver it to specialized vesicles for storage and eventual release. Reuptake via DAT is a major mechanism through which dopamine is cleared from synapses. Dopaminergic neurons in the substantia nigra of PD patients express higher levels of DAT (Uhl, 1998; Nass and Blakely, 2003) and greater DAT levels are linked to reduced dopamine turnover and smaller changes in synaptic dopamine concentration (Longo et al., 2017). This implies that an important functional role of DAT is to maintain relatively constant synaptic dopamine levels and to preserve dopamine in nerve terminals (Sossi et al., 2007, 2009; Lee et al., 2008).

The eight dopaminergic neurons of *C. elegans* have been fluorescently tagged with GFP using the DAT-1 promoter in neuronal transgenic strains BZ555 ([dat-1p::GFP]), BY200 [dat-1p::GFP, pRF4(rol-6(su1006)], and TG2435 ([dat-1p::GFP + rol-6(su1006)]) (Nass et al., 2002; Pu and Le, 2008; Masoudi et al., 2014; Cooper et al., 2015). Studying these cells in a genetic background of overexpressed α-synuclein or after treatment with the environmental toxin 6-OHDA has revealed that DA neurons degenerate with age and identified alleles that confer 6-OHDA resistance (Nass et al., 2005; Hamamichi et al., 2008).

The *C. elegans cat-2* gene encodes tyrosine hydroxylase, a rate-limiting enzyme for dopamine synthesis (Sulston et al., 1975; Omura et al., 2012; Masoudi et al., 2014). Overexpression of CAT-2 in *C. elegans* leads to age-dependent degeneration of dopaminergic neurons (Cao et al., 2005; Masoudi et al., 2014). Table 1 summarizes the most commonly used *C. elegans* strains for studying the molecular pathology and behavioral phenotypes of PD.

### Toxin-induced models

#### MPTP and 6-OHDA

In addition to transgenic *C. elegans* models involving the overexpression or mutation of PD-linked genes to study the genetic causes of PD, environmental agents have also been used to study PD-related neuronal degeneration and cell death (Nass et al., 2002; Pu and Le, 2008; Ali and Rajini, 2012; Zhou et al., 2013). Previous studies have modeled the motor aspects of PD using *in vivo* exposure to toxins that cause an overload of ROS and disrupt the electron transport chain in mitochondria leading to neuronal abnormalities and eventually cell death (Varcin et al., 2012; Dias et al., 2013; Hwang, 2013; Chege and Mccoll, 2014). The best studied neurodegeneration-inducing chemicals in *C. elegans* PD models are the toxins 6-OHDA (6-hydroxydopamine) and MPTP (1-methyl-1, 2, 3, 6-tetrahydropyidine) (Nass et al., 2001 Chakraborty et al., 2013; Chen P. et al., 2015).

MPTP was first identified as a PD-causing neurotoxin in humans in the 1980s after drug addicts in California inadvertently administered the agent in synthetic heroin (Langston et al., 1983). MPTP is highly lipophilic and can cross the blood brain barrier. In the brain, it is converted to 1-methyl-4-phenylpyridinium ion (MPP+) by glial monoamine oxidase B (Smeyne et al., 2005). MPP+ exerts neuronal toxicity by inhibiting complex I of the mitochondrial electron transport chain to induce mitochondrial dysfunction, decreasing the mitochondrial DNA content, and impairing autophagic degradation (Zhu et al., 2012; Miyara et al., 2016). Braungart and colleagues showed that wild-type *C. elegans* treated with 1.4 mM MPP+ at the L1 stage display developmental delays and exhibit an uncoordinated behavioral phenotype (twitcher and coiler) 3 days after treatment compared to untreated controls (Braungart et al., 2004). Further, MPP+ was actively taken up by the dopamine transporter and selectively degenerated dopaminergic neurons. In a screen that tested compounds for ameliorating the toxic effects of MPP+, two dopamine receptor agonists, lisuride and apomorphine, improved mobility and reduced coiling with no effect on development and mobility of wild-type animals, suggesting that improved symptoms resulted from the reduction of MPP+ toxicity (Braungart et al., 2004). Treating *cat-2::GFP* animals with 1.0 and 1.5 mM MPP+, degenerated dopaminergic neurons and led to reduced mobility (Braungart et al., 2004).

6-OHDA was first isolated in the 1950s (Senoh and Witkop, 1959; Senoh et al., 1959); it has a chemical structure similar to dopamine but with the addition of a hydroxyl group that makes it toxic to dopaminergic neurons (Blesa et al., 2012b). In PD research, the administration of 6-OHDA causes mitochondrial failure by inhibiting complex I of the mitochondrial electron transport chain. This results in ATP depletion and elevated oxidative stress, which ultimately leads to dopamine neuron damage (Glinka et al., 1997, 1998; Nass et al., 2002; Meredith et al., 2008; Pu and Le, 2008; Meredith and Rademacher, 2011; Ali and Rajini, 2012; Thiele et al., 2012). In *C. elegans*, 6-OHDA administration leads to the loss of GFP-labeled dopaminergic cell bodies and processes (Masoudi et al., 2014). Interestingly, two dopamine D2 receptor agonists, bromocriptine and quinpirole, ameliorate 6-OHDA toxicity in a dose-dependent manner via receptor-independent mechanisms (Marvanova and Nichols, 2007). CAT-2 overexpression confers resistance to 6-OHDA in wild-type and CAT-2 mutant backgrounds possibly due to reduced 6-OHDA uptake into dopaminergic neurons when excess dopamine is present (Masoudi et al., 2014). Due to the conservation between mammalian and *C. elegans* dopamine receptors, these and other results from toxin-induced neurodegeneration studies in *C. elegans* may help shed light on novel mechanisms leading to dopaminergic neuroprotection (Chen Y. M. et al., 2015).

#### Insecticides and herbicides

Rotenone (a broad spectrum insecticide), paraquat (an herbicide), and several other insecticides have been used to induce PD-like pathology in *C. elegans* (Ved et al., 2005; Settivari et al., 2009; VanDuyn et al., 2010, 2013; Jadiya and Nazir, 2012; Jadiya et al., 2012; Zhou et al., 2013; Gonzalez-Hunt et al., 2014). Both paraquat and rotenone trigger excessive ROS production in neurons, which leads to cellular damage (Ved et al., 2005; Miller et al., 2007; Tanner et al., 2010, 2011; Spivey, 2011; Zhou et al., 2013). *Caenorhabditis elegans* strains including BY250 (dat-1p:GFP), BZ555 (dat-1p:GFP), and UA57 ([dat-1p::GFP+dat-1p::cat-2]) can be exposed to these toxins to visualize and quantify abnormalities in neuronal morphology (Nass et al., 2002; Pu and Le, 2008; Liu et al., 2015; Li H. et al., 2016). Jadiya and colleagues selected specific neurotoxins to represent different pesticide classes including a botanical, an herbicide, a pesticide, a fungicide, an organophosphate, and a pyrethroid. The authors found that in strain NL5901 ([unc-54p::α-synuclein::YFP+unc-119(+)]), superoxide dismutase and heat shock protein genes exhibit a unique pattern of expression for each pesticide class (Jadiya and Nazir, 2012; Jadiya et al., 2012). In addition, rotenone significantly increased α-synuclein aggregation and oxidative stress, while reducing mitochondrial and lipid content in NL5901 animals (Jadiya and Nazir, 2012).

#### Manganese

Mn is an essential transition metal required for growth, development and cellular homeostasis (Prohaska, 1987; Takeda et al., 2003). It is a co-factor for multiple enzymes such as Mn superoxide dismutase, pyruvate carboxylase, arginase, and glutamine synthase, and can substitute for magnesium (Mg) in enzymatic reactions catalyzed by kinases (Horning et al., 2015). However, inhaling toxic levels of Mn can lead to nasal and pulmonary inflammation, renal dysfunction, and neurodegeneration (Aschner and Aschner, 1991). For example, occupational exposure through Mn mining, steel manufacturing, and welding are linked to increased risk for parkinsonian syndrome (Myers et al., 2003). Specifically, exposure to toxic levels of Mn can cause oxidative injury in the substantia nigra, the loss of dopaminergic neurons and phenotypes such as tremor, rigidity, and bradykinesia (Calne et al., 1994; Olanow, 2004). In *C. elegans*, Benedetto et al. found that extracellular and not intracellular dopamine is responsible for Mn-induced dopaminergic neurodegeneration, and that this process depends on a functional DAT-1 receptor and is linked to oxidative stress and lifespan reduction. Overexpression of the antioxidant transcription factor, SKN-1, reduces Mn toxicity, and dopamine-dependent Mn toxicity requires the NADPH dual-oxidase BLI-3. The authors proposed that *in vivo* BLI-3 (which has over 99% homology to the human DUOX genes) facilitates the conversion of extracellular dopamine into toxic reactive species, which get taken up by DAT-1 in dopaminergic neurons and cause oxidative stress and cell degeneration (Benedetto et al., 2010). Mn neurotoxicity was also studied in genetic DJ-1 models of *C. elegans* exposed to Mn (Chen P. et al., 2015); the results suggest that DJ-1 has a protective role and improves lifespan in Mn-exposed nematodes in an age-dependent manner.

## Markers of pathology in genetic and toxin-induced PD *C. elegans* models

### Alpha-synuclein expression

**Figure 1:**
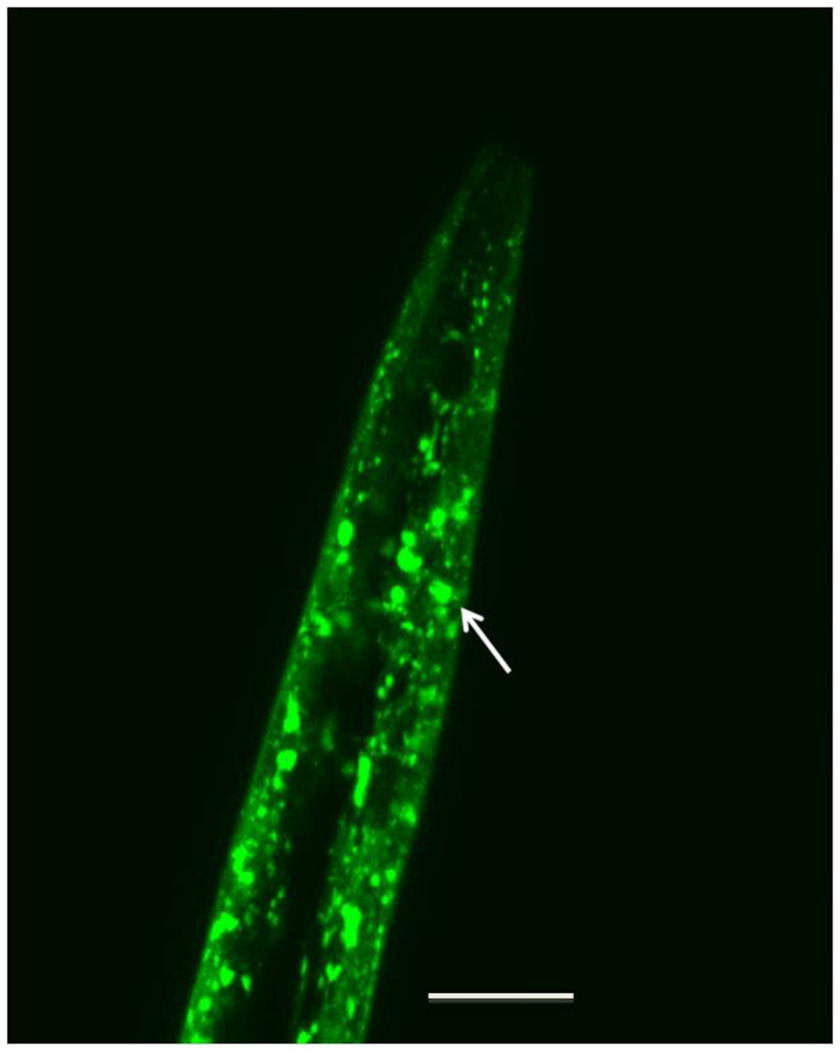
Representative image of the head region of a day-7 adult of strain NL5901 ([unc-54p::α-synuclein::YFP+unc-119]), maintained at 22°C, showing α-synuclein protein expression in the body wall muscle cells. The white arrow indicates one of multiple visible protein aggregates. Scale bar, 50 μm; magnification, 50×. (Original image taken by the authors for this paper on a Zeiss LSM 510 laser scanning confocal microscope).

To model α-synuclein aggregation and accumulation *in vivo*, researchers have generated transgenic *C. elegans* strains that express the human α-synuclein gene in body wall muscle cells and in neurons (Table 1). In these models, increased or decreased fluorescence intensity associated with YFP linked to α-synuclein can be quantified to determine the levels of protein expression (Jadiya et al., 2011; Jadiya and Nazir, 2012; Fatima et al., 2014; Fu et al., 2014a; Chen Y. M. et al., 2015; Liu et al., 2015). Loss of fluorescence intensity indicates reduced protein expression, whereas increased fluorescence indicates increased α-synuclein expression. Such changes in protein expression can be visualized using microscopy (Figure 1) and analyzed using freely available programs such as FIJI (Schindelin et al., 2012). Alterations in protein expression can also be assessed using techniques such as fluorescence resonance energy transfer (FRET) or fluorescence recovery after photobleaching (FRAP) (Bodhicharla et al., 2012).

### Neuronal morphology

**Figure 2:**
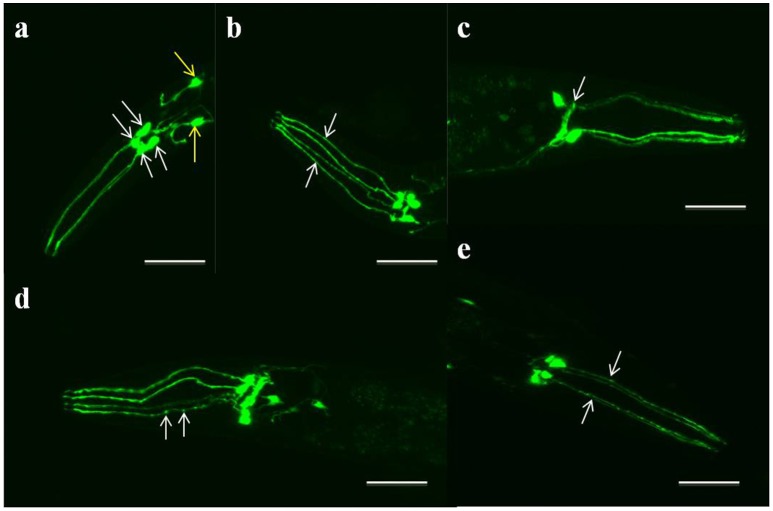
Representative images of the head regions of day-5 adults of strain BZ555 ([dat-1p::GFP]), maintained at 22°C. The images show **(a)** six healthy dopaminergic neurons (four CEPs [white arrows] and two ADEs [yellow arrows]), **(b)** two intact cell processes or dendrites (white arrows) of the four CEP neurons, which extend from the pharynx to the tip of the nose **(c)** the shrinkage of a cell body (white arrow), **(d)** neuritic blebbing (white arrows), and **(e)** abrupt gaps or breaks in the dendrites or cell processes (white arrows) caused by 50 mM 6-OHDA. (Scale bars, 50 μm; magnification, 50×. Original image taken by the authors for this paper on a Zeiss LSM 510 laser scanning confocal microscope).

Aberrant neuronal morphologies caused by exposure to neurotoxins or heavy metals can be visualized using fluorescence microscopy and quantified by counting the types and frequencies of aberrations. Such investigations typically focus on dopaminergic neurons of the head, i.e., the four CEPs and two ADEs (Figures 2a,b). Aberrant morphologies include the loss of neuronal cell bodies (Figure 2c), the absence of neuronal processes, broken neurites (Figure 2d), shrinking of dendritic endings, and the appearance of vacuoles (Nass et al., 2002; Pu and Le, 2008; Yao et al., 2010b; Masoudi et al., 2014). In addition, neurons exposed to toxins may appear dark, rounded and/or small, exhibit neuritic blebbing (Figure 2e), and lose GFP expression (Nass et al., 2002; Berkowitz et al., 2008; Pu and Le, 2008; VanDuyn et al., 2010; Ali and Rajini, 2012; Fu et al., 2014a; Masoudi et al., 2014). Selective degeneration can be scored based on any of these morphological changes or the absence of the neurons. In addition, *C. elegans* dopaminergic neurons that express human α-synuclein degenerate by mid-life (Hamamichi et al., 2008). In contrast, most genetic mouse models of α-synuclein fail to show degeneration of dopamine neurons (Blesa et al., 2012a; Blesa and Przedborski, 2014).

### Dopamine content

Dopamine reuptake transporters (DAT-1 in *C. elegans*) play a crucial role in the uptake of environmental toxins such as 6-OHDA and MPTP, which enter neurons, cause cell degeneration, and decrease the levels of endogenous dopamine (Gainetdinov et al., 1997; Nass et al., 2002; Pu and Le, 2008; Ali and Rajini, 2012; Masoudi et al., 2014). The dopamine content in *C. elegans* treated with a neurotoxin can be measured by reverse phase high performance liquid chromatography (RP-HPLC) with electrochemical detection (Pehek et al., 2005; Yao et al., 2010b; Satapathy et al., 2016). Reduced levels of dopamine and the resulting behavioral deficits are found in *C. elegans* overexpressing LRRK2 (both wild-type and mutated forms; Yao et al., 2010b). LRRK2 animals have a 50–72% reduction in dopamine levels compared to the wild-type control strain N2 (Yao et al., 2010b).

An alternative method is to measure dopamine content using HPLC followed by the detection of chemiluminiscence (Kuwahara et al., 2006; Tsunoda, 2006; Fu et al., 2014a). Post separation, colorimetric oxidation, fluorescence derivatization with ethylenediamine, and peroxyoxalate chemiluminiscence reaction detection are then performed on the extracts containing dopamine and its metabolites (Tsunoda, 2006). This method is highly sensitive, with detection limits in the fentomolar range, and makes it possible to measure dopamine in small-volume samples. HPLC with chemiluminiscence was used to show reduced dopamine levels and a reduced locomotory phenotype in transgenic *C. elegans* strains expressing A30P or A53T mutant α-synuclein in dopamine neurons (Kuwahara et al., 2006). In another study, the same technique revealed that the dopamine content of 6-OHDA-treated animals was 64% less than untreated controls (Fu et al., 2014a). Interestingly, the levels of dopamine in 6-OHDA-treated animals are elevated after treatment with the natural compound n-butylidenephthalide (Fu et al., 2014a). Additionally, using HPLC with UV detection, Ali and Rajini showed that the dopamine levels of MPTP and organophosphorous insecticide-exposed *C. elegans* are lower than in untreated controls (Ali and Rajini, 2012).

### Lipid content

**Figure 3:**
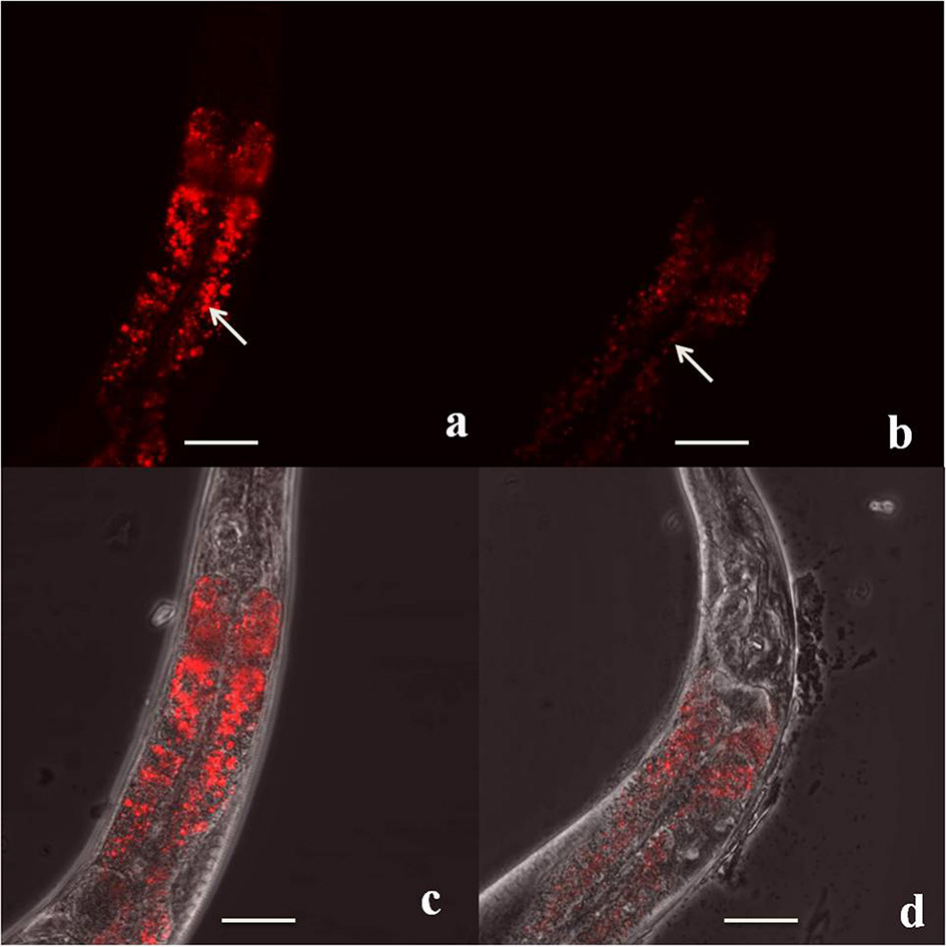
Representative images of Nile red staining of lipid content in live, day-5 adult *C*.* elegans* in **(a)** a wild-type N2 animal under fluorescence microscopy and **(b)** an OW13 [unc-54p::α-synuclein::YFP + unc-119(+)] animal under fluorescence microscopy. Image **(c)** depicts an overlay of phase contrast and fluorescence microscopy of a wild-type N2 animal and **(d)** shows an overlay of phase contrast and fluorescence microscopy of an OW13 animal. Strains were maintained at 22°C. White arrows represent stained fat droplets. Scale bar, 50 μm; magnification, 60x. Original image taken by the authors for this paper on an Olympus FLUORVIEW FV10i confocal microscope.

Prior research suggests that the aggregation of α-synuclein oligomers is associated with lipid peroxidation due to ROS overload, which can alter cellular membrane composition (Binukumar et al., 2010; Angelova et al., 2015). In *C. elegans*, intracellular fat droplets can be stained with the fluorescent dye Nile red (a lipophilic stain that fluoresces in a lipid environment), visualized with fluorescence microscopy (Figure 3), and quantified by analyzing the fluorescence staining. Several studies have shown that the NL5901 and OW13 strains have lower Nile red fluorescence than wild-type control N2 animals of the same age, indicating a reduced lipid content in PD strains (Jadiya et al., 2011; Jadiya and Nazir, 2012; Fu et al., 2014a). However, further investigation is warranted to describe the role of lipid content in *C. elegans* models of PD. For example, the lipid content of PD strains should be directly compared to their corresponding genetic controls, not just to wild-type N2 control strains. In addition, another widely used stain for measuring lipid content in *C. elegans*, Oil Red O, is a fat-soluble diazo dye that has been widely used to stain lipid droplets in mammalian cells and tissues, and has recently been applied to observing lipid stores in *C. elegans* (O'Rourke et al., 2009; Elle et al., 2010; Wahlby et al., 2014). This stain measures fat stores contained only in lipid droplets, which correlate well with biochemically-measured lipids (total fatty-acid methyl esters). Whereas Nile red primarily stains acidic lysosome-related gut granules in live or fixed animals (Elle et al., 2010; Wahlby et al., 2014), Oil Red O shows a better correlation with triglyceride levels. Depending on the solvent, Oil Red O can also stain cellular structures in non-adipogenic cell lineages (Elle et al., 2010). Yen and colleagues showed that fixed, but not Nile red-fed (live) wild-type N2 animals reveal fat stores that match label-free coherent anti-Stokes Raman scattering (CARS) imaging or Oil Red O and Nile Red fixed imaging (Yen et al., 2010). Another study by Barros and coworkers compared different methods (Nile red, BODIPY, Sudan Black and Oil Red O) to study the effect of dopamine signaling on fat content in *C. elegans*. Results showed similarity between fixative based dyes (Sudan Black and Oil Red O) and vital dyes (BODIPY and Nile Red) with smaller measurable decreases for the vital dyes (Barros et al., 2014). It will be interesting to further elucidate the role of lipid content in cellular hallmarks of PD by using these additional tools.

## Behavioral phenotyping

In humans, movement is controlled by synergistic inputs from the neuronal networks located in the substantia nigra of the ventral midbrain (Groves, 1983). These nerve cells together form an intricate network of axonal processes that synapse with dendritic spines by innervating the basal ganglia (Pickel et al., 1981; Freund et al., 1984). Crosstalk between neurons of the substantia nigra and basal ganglia results in dopamine release, which plays a crucial role in modulating movement (Bernheimer et al., 1973; Lanciego et al., 2012). This modulation is lost in PD due to the loss or degeneration of the dopaminergic neurons in the substantia nigra, which leads to motor dysfunction (Greengard, 2001). In spite of advancements in our understanding of the pathophysiology of PD and the development of several dopamine-based therapies, the exact molecular mechanisms by which dysfunctional dopaminergic systems lead to movement impairments in PD are not fully understood. In addition, dopamine also has been implicated in other functions such as eye movement, motor planning, learning, motivation, and addiction (Wise, 2004; Schultz, 2007). These multiple roles of dopamine in a complicated nervous system pose several questions about its precise role in movement-related disorders like PD. In *C. elegans*, the dopaminergic system has been well described and found to have structural and functional similarities to that of humans (Duerr et al., 1999; Lee and Ambros, 2001; Nass and Blakely, 2003; Suo et al., 2003; Chase et al., 2004; Chase and Koelle, 2007). In addition, some of the mechanisms of dopamine synthesis, storage, and transport in humans are conserved in *C. elegans*, and the nerve endings of dopaminergic neurons and synaptic vesicles have similar dopamine levels to those in mammalian neurons (Fuxe and Jonsson, 1973; Bargmann, 1998; Chege and Mccoll, 2014). Studies have established that disrupting dopamine signaling can lead to behavioral phenotypic changes in *C. elegans* such as altered movement (Omura et al., 2012), defecation (Vidal-Gadea and Pierce-Shimomura, 2012), egg-laying (Weinshenker et al., 1995), food sensing (Sawin et al., 2000), and response to external environmental cues including ethanol and nonanol (Lee et al., 2009; Kimura et al., 2010). In *C. elegans*, dopamine also controls acclimatization to mechanical stimuli (Sanyal et al., 2004), foraging (Hills et al., 2004), and transitions between crawling and swimming behavior (Vidal-Gadea et al., 2011).

### Basal slowing or food-sensing behavior

The locomotion rates of *C. elegans* change in the presence or absence of food, and feeding status. For example, well-fed, wild-type animals move more slowly in the presence of bacteria versus when there is no bacterial food source on the petri dish. This foraging behavior is dependent on dopaminergic neurons, which mechanically sense the presence and availability of bacterial food by its texture, and when food is present, decrease the animals' locomotion (Sawin et al., 2000). This slowing in response to abundant food in well-fed animals is called basal slowing. Deficits in dopaminergic function are associated with higher locomotion in the presence of food in well-fed animals, as evidenced by their lower basal slowing response (Yao et al., 2010b; Chen et al., 2013). Starved *C. elegans* slow down more dramatically in the presence of food, a phenomenon referred to as the enhanced slowing response, which ensures that the animals do not leave their newly found food source (Sawin et al., 2000; Rivard et al., 2010). Unlike basal slowing, which is controlled by dopaminergic neurons, the enhanced slowing response is regulated by serotonin (Sawin et al., 2000).

To determine the basal slowing response, animals are washed in buffer (typically M9) and then transferred to NGM plates with or without OP50 bacterial lawns. Basal slowing, which is measured as the frequency of body bends, is then recorded for 20–60 s and analyzed using data acquisition software as follows: (basal slowing = [rate of movement in the absence of food − rate of movement in the presence of food]/rate of movement in the presence of food) (Cooper et al., 2015). Basal slowing or food sensing behavior can also be measured as: (basal slowing = [movement rate of the animals in the presence of bacteria/movement rate in absence of bacteria] × 100) (Kuwahara et al., 2006). The study by Cooper et al. used food-sensing behavior to assess the functional loss of dopamine neurons in *C. elegans* expressing the familial Parkinson mutant human α-synuclein in dopamine neurons. The results showed that *C. elegans* expressing either human mutant α-synuclein (A53T) or human mutant LRRK2 (G2019S) exhibited deficits in this dopamine-related behavior (Cooper et al., 2015). This deficiency can be rescued by a mutation in the insulin-IGF1 receptor *C. elegans* ortholog, *daf-2*, a key modulator of aging pathways (Kenyon et al., 1993). Interestingly, the overexpression of LRRK2 (both wild-type and G2019S mutated forms) and *cat-2* deletion disrupt the age-dependent basal slowing response. This diminished behavior can be rescued by treatment with exogenous dopamine (Yao et al., 2010b; Johnson et al., 2015).

### Area-restricted searching (ARS) behavior

Area-restricted searching (ARS) is a foraging behavior in which wild-type animals minimize searching in areas that have abundant food and extend the search to larger areas when food is scarce. As the time since removal from food increases, animals turn less frequently towards the food (Hills et al., 2004; Gray et al., 2005; Chen et al., 2013). This is a goal-directed behavior that involves dopamine signaling. Removing or damaging dopaminergic neurons can lead to abnormal or abolished ARS behavior. For example, ARS behavior was rescued by administering exogenous dopamine to animals with defective dopamine signaling (Hills et al., 2004). ARS can be measured by transferring well-fed animals to NGM plates and videotaping them for 60 s after 5 and 30 min. The number of turns that exceed 90 degrees are counted from the tracks of each animal at each time-point (Cooper et al., 2015). ARS is impaired in both α-synuclein and LRRK2 PD mutants (Cooper et al., 2015). *Daf-2* mutations increase searching in both PD strains, suggesting a role for aging in modulating dopamine-dependent behaviors in nematode models of PD (Cooper et al., 2015).

### Chemotaxis assay

*Caenorhabditis elegans* can sense and respond to a multitude of environmental cues. These responses can be both aversive and attractive (Bargmann, 2006). For example, under standard laboratory culturing conditions, untreated wild-type (N2) animals avoid ethanol. However, when these animals are continuously exposed to ethanol, they develop a tolerance to and preference for ethanol, a response which is controlled by the dopamine system (Davies et al., 2004; Lee et al., 2009). Unlike wild-type animals, *cat-2* and *tph-1* mutants lacking a functional dopamine system do not develop an ethanol preference to chronic ethanol exposure (Lee et al., 2009). Ethanol avoidance is significantly decreased in non-ethanol-pretreated animals that express human mutant α-synuclein and mutant LRRK2 compared to those expressing wild-type α-synuclein and wild-type LRRK2 (Cooper et al., 2015). Interestingly, ethanol avoidance is restored in an α-synuclein mutant with a deletion of the *daf-2* gene, indicating that slowing aging also slows PD symptoms. To assay ethanol preference as a surrogate measure of the dopamine system, animals are incubated on an ethanol plate and transferred to assay plates that are divided into equal quadrants. Ethanol is provided in two quadrants, and animals are allowed to move freely for 30 min; the time preference for the quadrants is scored during this time. A preference index (PI) is calculated as ([number of animals in the ethanol quadrants]–[number of animals in control quadrants])/the total number of animals tested (Lee et al., 2009). This assay could also be used to assess the PI of PD animals with an impaired dopaminergic system caused by chemical exposure.

In *C. elegans*, the response to the aversive odorant nonanol is regulated by dopamine signaling (Bargmann, 2006; Kimura et al., 2010; Fatima et al., 2014; Sashidhara et al., 2014; Satapathy et al., 2016). When a drop of nonanol is placed near the head of a wild-type worm, the worm senses it and moves away as a chemotactic “aversive” response. However, when its dopamine content is diminished, the animals take longer to respond to the chemical stimulus. The response time to nonanol is increased 2-fold in the α-synuclein overexpressing strain NL5901 after treatment with *ida-1* (ortholog of mammalian diabetes autoantigen IA-2) RNAi (Fatima et al., 2014). In contrast, certain botanical compounds have shown to reduce the time required by both wild-type (N2 exposed to 6-OHDA/pesticide) and α-synuclein overexpressing strains (NL5901) to respond to nonanol (Sashidhara et al., 2014; Satapathy et al., 2016). This suggests that the “nonanol repulsion assay” can be used as an indirect measure of dopamine content in nematodes with impaired dopamine signaling.

### Swim to crawl transition

Gait can be defined as alterations in the patterns of movement based on the environment currently occupied by an animal. In humans, the basal ganglia regulate motor movement during gait, which activates dopaminergic neurons (Marsden, 1982; Mink and Thach, 1991; Fukuyama et al., 1997; Koepp et al., 1998). In PD, dysfunction in the basal ganglia region contributes to impaired gait functions and rhythms (Morris et al., 1996; Hausdorff et al., 1998; Sofuwa et al., 2005). Gaits in *C. elegans* are mainly characterized as crawling (on solid “agar” media) and swimming (in liquid media) (White et al., 1986; Pierce-Shimomura et al., 2008). On agar, nematodes move or crawl in a classical sinusoidal fashion. This changes to “thrashing” or swimming when the animals are moved to liquid media. The mechanisms behind this gait transition are unknown; however, roles for bioamine neurotransmitters such as dopamine and serotonin have been implicated (Mesce and Pierce-Shimomura, 2010).

In *C. elegans*, dopamine is responsible for a wide array of behaviors including the gait transition from swim to crawl (Vidal-Gadea et al., 2011). The activation of dopamine neurons by optogenetics confirmed that the switch from crawling to swimming involves signaling through D1-like dopamine receptors, which is similar to the pattern the animals exhibit when they crawl off the bacterial food source (Sawin et al., 2000; Vidal-Gadea et al., 2011). Under both conditions, dopamine functions by decreasing the speed of the animal's movement. Animals with impaired dopaminergic signaling can exhibit opposing behavioral phenotypes, and the genetic ablation of all dopaminergic neurons can impair the transitions between swimming and crawling and lead to paralysis in animals due to incessant swimming (the swimming-induced paralysis or SWIP phenotype; Vidal-Gadea et al., 2011). The dopamine transporter DAT-1 plays an important role in dopamine reuptake and clearance. In animals that exert maximal physical activity during swimming, mutations in DAT-1 lead to SWIP (McDonald et al., 2007). In humans, PD is characterized by impaired gait and the failure to transition between locomotory patterns (Jankovic, 2008). The failure of *C. elegans* to transition between swimming and crawling when the dopamine system is impaired reinforces the validity of *C. elegans* PD models.

A swim-to-crawl assay involves growing animals on NGM plates seeded with OP50 bacteria and then changing the environmental conditions to affect movement. Such changes could include increasing or decreasing the viscosity of the medium or providing mechanical stimulation with magnetic particles, as described by Vidal-Gadea et al. (2011). Gait transitions are evaluated by video recording movement before and after altering the conditions. For swim-to-crawl transition, *C. elegans* lacking dopaminergic neurons will exhibit truncated movement upon transitioning from a liquid to an agar medium. Similarly, animals lacking the DAT-1 receptor accumulate high amounts of endogenous dopamine, which induces a switch from the swim to crawl phenotype before causing the SWIP phenotype (McDonald et al., 2007; Vidal-Gadea et al., 2011). In *C. elegans*, the swim-to-crawl assay has been used to demonstrate that the membrane protein tetraspanin (TSP-17) protects dopaminergic neurons against 6-OHDA-mediated neurodegeneration and the toxicity caused by increased concentrations of endogenous intracellular dopamine (Masoudi et al., 2014).

### Mechanosensory responses

Previous studies in *C. elegans* indicate that dopaminergic neurons are mechanosensory (Loer and Kenyon, 1993; Liu and Sternberg, 1995; Duerr et al., 1999; Sawin et al., 2000; Bettinger and McIntire, 2004; Hills et al., 2004; Sanyal et al., 2004; Abdelhack, 2016). Dopaminergic neurons respond to anterior touch stimulation (Sanders et al., 2013). *Caenorhabditis elegans* lacking tyrosine hydroxylase (*cat-2* mutants) display defective food-sensing behavior because they fail to slow down when they encounter a bacterial food source. This basal slowing response is mediated by dopamine signaling and depends on physically touching the bacterial food source (Sawin et al., 2000). Such interactions between dopamine and mechanosensory touch responses are not well understood. Nevertheless, these interactions appear to be necessary for regulating foraging in nematodes (Sawin et al., 2000). This confirms a role for dopamine in modulating the response to a non-localized mechanical stimulus (such as taps) administered to the NGM plate (Sanyal et al., 2004). Animals respond to external tapping by escalating their forward or backward motion. Repeated tapping attenuates the reversal frequencies and leads to habituation (Rose and Rankin, 2001). The time required to respond to the tap can be used as a measure of dopaminergic function as the loss of dopaminergic function can alter this behavior (Sanyal et al., 2004). Although this behavior has not yet been studied in animals with Parkinson's-like symptoms, mechanosensory touch responses have been studied in *C. elegans* neurodegenerative models of Huntington's disease, Alzheimer's disease, and tauopathies (Parker et al., 2001; Miyasaka et al., 2005; Gordon et al., 2008). Therefore, this behavior can be used to assess healthy/impaired dopaminergic function in wild-type and PD animals using *cat-2* mutants as a negative control, since these mutants habituate to tapping faster than wild-type strains (Chen et al., 2013).

### Dauer-dependent behavior

Under favorable conditions, the life cycle of *C. elegans* includes the egg stage, four larval stages (L1-L4), and an adult stage, which is reproductive in hermaphrodites and lasts for 3–5 days. When exposed to overcrowded conditions, limited food, or chemical or physical stressors, animals enter an alternative stage after L2 known as dauer diapause (Cassada and Russell, 1975; Fielenbach and Antebi, 2008). The entry to dauer is regulated by *daf-16* (forkhead box O or FOXO) and its upstream regulator *daf-2* (insulin receptor), which are important modulators of aging and lifespan (Kenyon et al., 1993; Lee et al., 2001). Although this behavior occurs independent of dopamine signaling, once the animals enter the arrest phase they respond to any changes in dopamine signaling by increasing their body movement (Gaglia and Kenyon, 2009). Therefore, dauer movement assays can be used to assess this behavioral change. For example, dauer formation can be induced by exposing *djr-1.2* mutants to the heavy metal Mn, transferring them to NGM plates without bacterial food, and storing for 72 h (Chen P. et al., 2015). The dauer diapause can then be determined using body movements which is defined as one complete body bend in forward or backwards direction in a 1 min duration (Gaglia and Kenyon, 2009; Chen P. et al., 2015). The *cat-2* deletion mutants that have diminished DA signaling are used as a positive control. Both *djr-1.2* and *cat-2* mutants exhibit increased dauer movement compared with controls. When exposed to Mn, the *djr-1.2* mutants show a further increase in movement compared with untreated controls, indicating reduced dopamine signaling (Chen P. et al., 2015). However, this behavior can be rescued by the overexpression of DAF-16. This behavioral assay was also important for assessing the interactions between aging, a PD environmental risk factor (i.e., Mn), and the PD-associated homolog DJ-1 (Chen P. et al., 2015).

### Fecundity

Fecundity is an important assay for determining the egg-laying behavior of *C. elegans* and is controlled by dopamine (Schafer and Kenyon, 1995; Weinshenker et al., 1995). The exposure to environmental toxins can lead to changes in dopamine signaling, which in turn can alter fecundity or brood size in *C. elegans* (VanDuyn et al., 2010). Fecundity can be measured by performing progeny count assays. Age-synchronous adults are placed on individual plates each day until they cease reproducing. The number of eggs or viable progeny is then counted. When the assays are performed by counting progeny, the plates are incubated at a specific temperature and the eggs are allowed to develop for 48 h before the brood size is determined (Hodgkin and Barnes, 1991; Scerbak et al., 2016). The assessment of brood size or total progeny has been performed in neurotoxin-treated (6-OHDA and insecticide) models (Satapathy et al., 2016) and in different PD mutants (Cooper et al., 2015) of *C. elegans*. LRRK2 mutants have decreased fecundity due to decreased levels of DA, and this decrease cannot not be rescued by the *daf-2* mutation (Yao et al., 2010b; Cooper et al., 2015). Also, a significant decrease in brood size (25–31%) occurs in animals exposed to 6-OHDA, which can be slightly increased by curcumin treatment (Satapathy et al., 2016). Overall, fecundity can be used to measure healthspan in wild-type and PD animals to assess the effects of experimental treatments on the overall pathology and behavioral phenotypes of *C. elegans*.

### Rate of defecation

In *C. elegans*, defecation is a behavior controlled by a series of muscle contractions, i.e., a motor program that occurs in the intestinal “enteric” muscles of the animals. On average, it occurs every 50 s (Dal Santo et al., 1999) and this cycle remains constant at 20°C. Dopamine has been implicated in controlling the defecation cycle (Weinshenker et al., 1995; McDonald et al., 2006; Vidal-Gadea and Pierce-Shimomura, 2012). Previous studies have demonstrated that excess dopamine reduces the defecation rate by decreasing expulsion muscle contractions (Weinshenker et al., 1995). Defecation is carried out in three steps: posterior body muscle contraction (pBoc), anterior body muscle contraction (aBoc), and expulsion muscle contraction (Branicky et al., 2001; Kwan et al., 2008). The length of the defecation cycle can be determined by viewing animals with a dissecting microscope and measuring the duration between two consecutive pBoc contractions in adult animals at 20°C or as specified (Branicky et al., 2001; Cooper et al., 2015). A recent study showed slower rates of defecation in α-synuclein and LRRK2 mutants compared to normal rates in *cat-2* mutants, suggesting that defecation behavior occurs independent of dopamine in these PD models (Cooper et al., 2015). However, *cat-2* mutants may not completely lack dopamine (Sanyal et al., 2004). The rate of defecation should be further investigated as an indicator of physiological outcome in PD animals.

### Locomotion

In *C. elegans*, locomotion or motility is a useful marker to assess healthspan (Bansal et al., 2015). The dorsal and ventral muscles coordinate to control the classical sinusoidal locomotion patterns in nematodes (Croll, 1975; Donnelly et al., 2013). Motility can be assessed in aged individuals using an A-B-C class-based system (Herndon et al., 2002). Class A represents a normal sinusoidal pattern, class B represents spontaneous reversals or induced motion with gentle prodding, and class C represents no movement or only movement of the head in response to gentle prodding. These patterns are also influenced by the presence or absence of food and exposure to mechanical or chemical stimuli (Omura et al., 2012). Studies have shown that disrupting DA signaling using genetic mutations or exposure to environmental toxins (6-OHDA or MPTP) can change the locomotory behavior of *C. elegans* (Ali and Rajini, 2012; Cooper et al., 2015; Liu et al., 2015). Such altered behavior can be assessed by observing changes in the typical sinusoidal pattern, including irregular body bends or thrashing behavior. Body bends are counted as one muscle contraction that leads to a complete bend of the dorsal or ventral side of the animal (Ghosh and Emmons, 2008). The term “thrashing” is used to define motility when nematodes are placed in a drop of liquid (e.g. M9 buffer), and it is determined by measuring the frequency of lateral movements or the direction of mid-body bending (Buckingham and Sattelle, 2009). Locomotory behavior can be quantified by viewing or recording worm movements through a stereomicroscope. Numerous automated programs facilitate the analysis of digitally recorded data, including Worm Tracker 2.0, OptoTracker, The Parallel Worm Tracker, Nemo, Multimodal illumination and tracking system, the Multi Worm Tracker, and CoLBeRT (Husson et al., 2013). Recently, a microfluidic device was also used to measure the locomotion of *C. elegans* using an electric signal (Jung et al., 2016).

## Conclusions

Well-developed imaging techniques and genetic malleability make *C. elegans* a useful model for testing compounds to treat the cellular and related behavioral symptoms of PD and investigating the basic molecular mechanisms underlying potential therapeutic approaches. The pathological and behavioral markers discussed in this review could be useful for performing screening experiments and establishing crucial connections between PD-like pathology, possible susceptibility factors, and the mechanisms triggered by exposure to novel drug molecules.

## Author contributions

MM and SM contributed equally to the concept, background research, and writing of the manuscript. EMV contributed to the background research, writing and editing of the manuscript. All authors made intellectual contributions, edited, and approved the manuscript for publication.

### Conflict of interest statement

The authors declare that the research was conducted in the absence of any commercial or financial relationships that could be construed as a potential conflict of interest.
